# Designing a novel method based on multiplex PCR for detecting various meat of birds in processed ground meat products

**DOI:** 10.1016/j.fochms.2023.100177

**Published:** 2023-07-13

**Authors:** Negin Rajaei, Abbas Doosti

**Affiliations:** a Department of Biology, Faculty of Basic Sciences, Shahrekord Branch, Islamic Azad University, Shahrekord, Iran; b Biotechnology Research Center, Shahrekord Branch, Islamic Azad University, Shahrekord, Iran

**Keywords:** Multiplex PCR, Bird’s meat species, Validation

## Abstract

Falsified food directly influences wildlife, fair trade, religion, and the health of society. Here, we report a multiplex polymerase chain reaction to evaluate the accurate determination of seven species of bird meat in meals on a single assay platform. To amplify segments of DNA from Columba livia, Corvus moneduloides, Gallus gallus, Coturnix japonica, Phasianus colchicus, Struthio camelus, and Meleagris gallopavo meats, respectively, a total of seven sets of species-specific primers targeting the mitochondrial and cytochrome *b* genes were developed. Gel photographs and electrochromatography from an Experion Bioanalyzer were used to identify all PCR products. Species specificity checks discovered no cross-species amplification. The applicability of its screening to find target species in processed food was shown in commercial and model meatballs. A validation study revealed that the test is reliable, quick, affordable, repeatable, specific, and accurate down to 50,000 mitochondrial copies. It might be used for raw meats and products involving processed and severely deteriorated food samples. The customers, the food business, and law enforcement would all benefit immensely from this suggested approach.

## Highlights

• A reliable and sensitive assay was developed to verify meat adulteration in the food chain.
• The multiplex PCR system was optimised for the identification of *Columba livia, Corvus moneduloides, Gallus gallus, Coturnix japonica, Phasianus colchicus, Struthio camelus, and Meleagris gallopavo* DNAs in a single reaction mixture simultaneously.
• The sensitivity of the prepared kit for different meat samples was determined to be 50,000 copies/microliter to avoid false negative results.
• The technique developed here can be easily used for screening different birds' meat products for export and import purposes as well as for food inspection and diagnostic laboratories.

## 1 Introduction

Food adulteration, which negatively affects the product's quality, is commonly defined as the replacement of all or some of the food's constituents with inferior or less expensive ones (Bansal et al., 2017). Two forms of food adulteration may be distinguished: accidental adulteration, which results from negligence, ignorance, or a lack of adequate facilities, and purposeful adulteration, which is a choice made with the aim to increase profits (Agarwal et al., 2023). A horse meat scandal in 2013 that included the mixing of horse meat with beef patties in certain regions of Europe brought attention to contaminated meals all around the globe. Food adulteration has grown to be a major issue with rising financial and health implications (He et al., 2021). Food safety and quality control depend on the efficient and accurate determination of food authenticity (Beć et al., 2022).

To obtain a competitive edge or increase profits, substituting pricey meat with less expensive meat has grown widespread along with the rise in consumption of meat in both emerging and developed nations (Österblom et al., 2022). The accuracy of the meat species information on food product labels is crucial for consumer education (Aprile and Punzo, 2022). Evaluation of the validity of the meat product label is essential due to the rise in allegations of adulteration of meat products (Grundy et al., 2022).

Along with beef and pig, bird meat, including chicken, are the main meat eaten in many nations. Bird meat is often less expensive than red meat and readily accessible (Asadipour et al., 2023). Furthermore, even though bird meat is lean, its protein composition is comparable to that of other meats (Piri-Gharaghie et al., 2023). As a result, poultry is one of the most consumed meats, and there are several dishes made with poultry across the globe. Meat from mechanically separated birds is commonly used in processed goods because it offers a cheap way to get protein (Hussain et al., 2015). Therefore, it's possible that red meat was mistakenly or fraudulently changed for avian meat (Karcher and Mench, 2018); this emphasizes the need of developing techniques to detect the species of meat in tainted food, particularly in minced meat and homogenized meat products (Ferone et al., 2020).

There has been an increasing need for the development of techniques that may rapidly and correctly identify the adulteration on site in cases of possible food adulteration (Ndudzo et al., 2022). Additionally, the food sector is looking at detecting techniques that may be used outside of laboratories since it must adhere to food-labeling compliance (Loeffler et al., 2022). The approach must be easy to use, quick, precise, and sensitive for on-site detection (Umapathi et al., 2022). Additionally, the analytical tools must be easy to move and carry (preferably in the hand), and the outcomes must be obvious (Zarinnezhad et al., 2021). The field must be able to use the on-site approach, from sample preparation through outcome verification. Recently, a lot of research has been done on the on-site identification of genes in food (Qin, 2019).

Due to recent developments in packaging technology, it is currently difficult or impossible to identify food components based on physical features (Jeevahan et al., 2020). The current techniques for determining the authenticity of meat depend on lipid, protein, and nucleic acid indicators (Piri Gharaghie and Sadat Shandiz, 2018). Lipid-biomarkers, which contain both the sort and amount of lipids, may experience significant changes during the preparation of meals, whereas protein-biomarkers are vulnerable to physio-chemical shocks (Finkel et al., 2023). However, tiny DNA biomarkers, in particular, are very robust in a deteriorated environment. Using DNA-based approaches, identifying species in animal meals and feeds has become more critical in recent years (Adenuga and Montowska, 2023).

Because it offers the potential for simple, rapid, specific, and sensitive evaluation, allowing for species detection even in complex and processed foods, the polymerase chain reaction (PCR) approach using species-specific primers is frequently used (Alikord et al., 2016). Multiplex PCR amplifies various target regions by mixing many primers, a promising technique for classifying meat species. One of the PCR-based detection methods, multiplex PCR, has recently been used to locate animal DNA (Adenuga and Montowska, 2023). Numerous DNA-based techniques have been reported for meat species authentication, including Multiplex-specific PCR, PCR-RFLP, PCR-based product DNA sequencing, real-time PCR, and DNA barcoding. It is the most efficient and trustworthy when comparing Multiplex-specific PCR to other methods like DNA barcoding, PCR-RFLP, PCR-RAPD, and single nucleotide polymorphism (SNP) analysis (Galal-Khallaf, 2021, Piri Gharaghie et al., 2022a).

Using a well-designed species-specific PCR under ideal conditions to identify and recognize species eliminates the need for restriction digestion and/or PCR product sequencing. However, since they allow multiple target detection on a single test platform while saving time and money, multiplex PCR assays utilizing species-specific primers have more significant potential than conventional single-species PCR methods. Much multiplex PCR (PCR) procedures have been documented for determining the identity of various animal species. However, they have not been created to authenticate prohibited species in bird base meals. The base meat-producing organisms of birds in raw and cooked meats and industrial meat products may now be identified for the first time using a multiplex PCR test that we developed.

## 2 Materials and methods

### 2.1 Meat sample collection

*Columba livia, Corvus moneduloides, Gallus gallus, Coturnix japonica, Phasianus colchicus, Struthio camelus, and Meleagris gallopavo* were the target meat species; meat samples from these species were collected from the Faculty of Veterinary Science at the Islamic Azad University of Shahrekord, Iran. Meats from *Coturnix japonica* and *Phasianus colchicus* were purchased from Fadak Farm in Isfahan, Iran (n = 50 each). *Meleagris gallopavo* (n = 50) and *Struthio camelus* (n = 50) were bought from the Mehregan ostrich farm in Isfahan (Iran). The food composition of these animals was prepared from the products of Faradaneh company (Faradaneh, Shahrekord, Iran). Additionally, within three days, the most popular commercial meats (n = 50) were bought from different stores in Shahrekord, Iran. All meat specimens and products were broken up into little pieces (50 gr) and transported in ice-chilled conditions (4 ˚C) and frozen at −20 ˚C until usage to avoid natural and enzymatic degradation of meats and DNA.

### 2.2 Preparation of meatballs

Two different kinds of meatballs were made following Rahman et al. (2014). The first category of beef meatballs was made by individually adding 1% of each target meat species. In contrast, the second kind included all objective meat species to generate a beef meatball that was 5% adulterated overall. Each meatball was created using 90% ground meat/meats emulsified (target meat species and beef) and 10% starch (Parsian BioProducts, Shahrekord, Iran), and the appropriate amounts of salt and seasonings like pepper, garlic, onion, etc (Faradaneh, Shahrekord, Iran). To imitate lengthy cooking and boiling effects, meatballs were autoclaved for 2.5 h at 121 ˚C under 45 psi pressure (Hastaran-Teb, Tehran, Iran). Before DNA extraction, all samples were produced in triplicate on three separate days by three different analyzers, and they were all kept at −20 ˚C. Three separate halal-branded beef meatballs (identified as A-C) were purchased, and model meatballs of pure and intentional contaminations were created in the lab.

### 2.3 DNA extraction

Using the Cinna Colon Genomic DNA Kit (Cinna Colon Co., Ltd., Tehran, Iran) and 20 mg of raw meat tissues, all meat samples' total DNA was extracted by the manufacturer's instructions. A 50 mg sample was used to extract DNA from commercial meat products. The material was first ground into a pulp in a 1.5 ml micro-centrifuge vial using a Micropestle, and then 20 µl of Proteinase K (Cinna Colon Co., Ltd., Tehran, Iran) was added. The mixture was incubated at 60 ˚C for 30 min to lyze the sample. To ensure the sample lysate was clear, the mixture was incubated once more at 60 ˚C for 20 min after adding 400 µl of lysis buffer (Cinna Colon Co., Ltd., Tehran, Iran). The following stages were carried out following the ions provided by the kit's maker, Cinna Colon Co., Ltd. of Iran. A UV–Vis Spectrophotometer (Libra S80, Biochrom, England) was used to assess the concentration and purity of the isolated DNA.

### 2.4 Primer design for multiplex PCR

Since mitochondrial genes are well protected by the mitochondrial membrane, maternally inherited, and present in multiple copies per cell, species-specific primers have been generated to target the mitochondrial genes of *Columba livia, Corvus moneduloides, Gallus gallus, Coturnix japonica, Phasianus colchicus, Struthio camelus,* and *Meleagris gallopavo*. For most animals and plants, the mitochondrial genes provide an adequate target length, sufficient conserved areas between species and across species, and an accessible sequence database. Contrarily, the *cytochrome b (cytb)* gene is an excellent candidate to research phylogenetic development at the intra- and inter-species stages and a target for particular primers and probes due to its modest evolutionary pace and distinct evolutionary patterns. These characteristics increased our desire to create species-specific primers targeting *the cytochrome b (cytb)* gene for the *Phasianus colchicus* species. *Columba livia* (MW487992), *Corvus moneduloides* (NC_051471), *Gallus gallus* (OL689235), *Coturnix japonica* (MW574361), *Phasianus colchicus* (MT841126), *Struthio camelus* (NC_002785), and *Meleagris gallopavo* (EF153719) whole genome sequences were acquired from the NCBI database (https://www.ncbi.nlm.nih.gov/) and were aligned utilizing ClustalW sequence alignment tool (https://www.ebi.ac.uk/Tools/msa/clustalw2/) (Wellcome Genome Campus, Hinxton, Cambridgeshire, UK) to specify the inter-species hyper-variable and intra-species conserved areas. MEGA5 software (https://www.megasoftware.net/) was used to verify mismatches to all other species, either at the 30 positions or, where feasible, for both forward and reverse primers. Utilizing the online BLAST local alignment tool in the NCBI database (https://blast.ncbi.nlm.nih.gov/Blast.cgi), the chosen primers were also checked for unique specificity to exclude cross-species binding with other animal or plant species. Cinna Colon (Cinna Colon Co., Ltd., Tehran, Iran) provided the intended primers for purchase.

### 2.5 Multiplex PCR amplification

All samples were produced and amplified using the PCR (Bio-Rad, USA) dilution protocol. To prepare each sample, 20 µl of phosphate-buffered saline (PBS) (Parsian BioProducts, Shahrekord, Iran) was added, and the mixture was heated at 98 ˚C for 2 min. A 1.5 ml sample from every meat pre-PCR dilution was combined to create the mixed meat sample. The PCRs were carried out in a total volume of 30 µl and contained 7 µl (0.5 of each) primer pair (Table 1), 5 µl sterile water, 3 µl DNA pool, and 15 µl master mix PCR Buffer (Ampilicon, Denmark) including 1.0 unit Phire Hot Start II DNA Polymerase. Amplifications were carried out using a T100TM Bio-Rad thermal cycler with the following PCR conditions:

**Table 1:** The list of primers was designed in this study.

Type of meat	Target gene	ACCESSION No	Sequence	Size (bp)	Annealing TM (˚C)
*Columba livia*	Mitochondrial	MW487992	F: 5′- AACTTCATCACAACTGCCATTAAC-3′ R: 5′- GTTTAGGTTTCGGTCTGTGAGC-5′	162	60˚ C
*Corvus moneduloides*	Mitochondrial	NC_051471	F: 5′- CGACCTTGCTATTTTCTCACTACAC-3′ R: 5′- ATGTGGTATTGAGGTTTCGGTC-3′	227	60˚ C
*Gallus gallus*	Mitochondrial	OL689235	F: 5′- ACTCTTTACCTAATTTTCGGCAC-3′ R: 5′- AAGATGGCTAGGTCTACTGATGC-3′	395	60˚ C
*Coturnix japonica*	Mitochondrial	MW574361	F: 5′- AGTCCTCATCACCGCTATCTTAC-3′ R: 5′- TAGTATGGGCGGATCTCATTTG – 3′	457	60˚ C
*Phasianus colchicus*	Cytochrome *B*	MT841126	F: 5′- CATACATTACACCGCAGATACCTC-3′ R: 5′-TTTGTCGGAGTTGGATGAGATG-3′	496	61˚ C
*Struthio camelus*	Mitochondrial	NC_002785	F: 5′- ACCTCTTCTGATTCTTCGGACAC-3′ R: 5′- AGTGTGCTTTTGCTCATGTCG-3′	543	61˚ C
*Meleagris gallopavo*	Mitochondrial	EF153719	F: 5′- GAGACCCAATCCTATATCAACACC-3′ R: 5′- CCTAAGAAGTGTTGTGGGAAGAAG-3′	619	61˚ C

Initial denaturation at 95 ˚C for 5 min; 40 cycles of denaturation at 95 ˚C for 20 s; annealing at 60 ˚C for 20 s, extension at 72 ˚C for 25 s; and a final extension at 72 ˚C for 5 min. Until further examination, the PCR products were kept at 4 ˚C. To check for contamination, a negative control was added to each set of responses. The test was repeated 3 times.

### 2.6 Agarose gel electrophoresis

Electrophoresis on a 1% low melting point gel was used to differentiate between the PCR products. Ethidium bromide (Parsian BioProducts, Shahrekord, Iran) was used to stain the PCR products, and electrophoresis was carried out at 100 V for 30 min. The gels were examined and photographed using a Bio-rad GelDoc 1000 gel recording device (Bio-rad, USA). The test was repeated 3 times.

### 2.7 Analyzing repeatability within a single LOT and different LOTs

The repeatability of the method was determined on the same day. Using kits produced by a single LOT, two users assessed 20 positive controls. The correlation rates of several LOTs were assessed. Reproducibility was assessed using data from two different lots. Twenty positive controls were examined using kits produced in two LOTs. The test was repeated 3 times.

### 2.8 Multiplex PCR assay validation

The specificity, reproducibility, sensitivity, and robustness of the multiplex assay used in this work were validated (food product testing). All seven target species and potential domestic and wild meat species that may be eaten in Iran were cross-tested with the assay for the specificity test. 14 voucher-target meat specimens from various suppliers were examined using the assay for the reproducibility assessment. Quantified mitochondrial replicas of the seven target species were created for the sensitivity test. The test was used to amplify five concentrations (100,000, 50,000, 25,000, 12,500, and 6250 mitochondrial copies/µL) in order to establish the minimum number of mitochondria that may be identified. The created assay analyzed food items for robustness and real-world performance. The test was repeated 3 times.

### 2.9 Statistical analysis

GraphPad Prism 5.0 (https://www.graphpad.com/support/prism-5-updates/) was used to examine the data and perform statistical tests. A one-way analysis of variance (ANOVA) was used to compare means, followed by a Tukey– Kramer post hoc test with a 95 percent confidence interval. Differences were considered significant at *p* < 0.05.

## 3 Results

### 3.1 The extraction of DNA results

Optical density (O.D.) measurements at 260 nm were used to estimate the amount of total extracted DNA. The DNA quality was determined by measuring the ratio between O.D. measurements at 260 nm and 280 nm employing a Biochrom Libra UV–Vis Spectrophotometer (Biochrom, Cambridge, United Kingdom). All specimens had high-quality DNA, as shown by the OD260/OD280 values of extracted DNA, which ranged from 1.7 to 2 (Fig. S1 A).

### 3.2 Single-plex PCR amplification

**Fig. 1:**
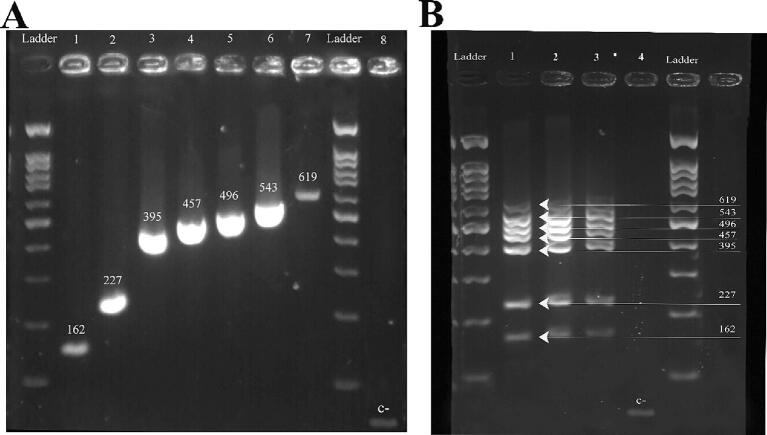
**A)** Seven target species isolated on a 2% agarose gel were amplified ly in a single-plex. Ladder: 100 bp, 1: *Columba livia* (162 bp), 2: *Corvus moneduloides* (227 bp), 3: *Gallus gallus* (395 bp), 4: *Coturnix japonica* (457 bp), 5: *Phasianus colchicus* (496 bp), 6: *Struthio camelus* (543 bp), 7: *Meleagris gallopavo* (619 bp), and 8: negative control. All lanes showed the anticipated PCR products. **B)** Multiplex PCR amplification of 7 target species. 1: *Columba livia*, *Corvus moneduloides*, *Gallus gallus*, *Coturnix japonica*, *Phasianus colchicus*, *Struthio camelus*, and *Meleagris gallopavo* raw meat, 2: Beef meatballs were made by individually adding 1% of each target meat species. 3: All objective meat species to generate a beef meatball that was 5% adulterated overall. 4: negative control.

First, meat samples were used to perform singleplex PCR amplification with each of the seven species-specific primers employed in this study. This was done to test the primers' effectiveness and specificity and determine whether PCR could be used to authenticate meat. According to the findings, raw meat samples could successfully be used for PCR to identify the meat species. Each species-specific primer pair caused the anticipated PCR products of 162, 227, 395, 457, 496, 543 and 619 bp for *Columba livia*, *Corvus moneduloides*, *Gallus gallus*, *Coturnix japonica*, *Phasianus colchicus*, *Struthio camelus* and *Meleagris gallopavo*, respectively. In the negative controls, there was no PCR product to be seen (Fig. 1A). When using sterile water further to dilute the pre-PCR solution (1:10 to 1:100), the issue of activation from a large sample size (>1 mm^2^) was occasionally solved (Fig. S1B).

### 3.3 Multiplex PCR amplification

The simple multiplex test for the simultaneous identification of the seven meat species has been established effectively. Fig. 1B displays seven different PCR fragments of various sizes according to the predicted sizes of the seven target meat organisms. The negative control did not produce any PCR fragments.

### 3.4 Validation of multiplex PCR assays

#### 3.4.1 Specificity assessment

Seven target species (*Columba livia, Corvus moneduloides, Gallus gallus, Coturnix japonica, Phasianus colchicus, Struthio camelus, and Meleagris gallopavo*) were analyzed using the developed multiplex PCR assay to test the assay's specificity. The outcome demonstrated that the test was particular to the targets: the anticipated PCR fragments were exclusively produced from the target species as predicted, and no evidence of a cross-reaction from non-target species was discovered.

#### 3.4.2 Reproducibility of the Multiplex-PCR method

**Fig. 2:**
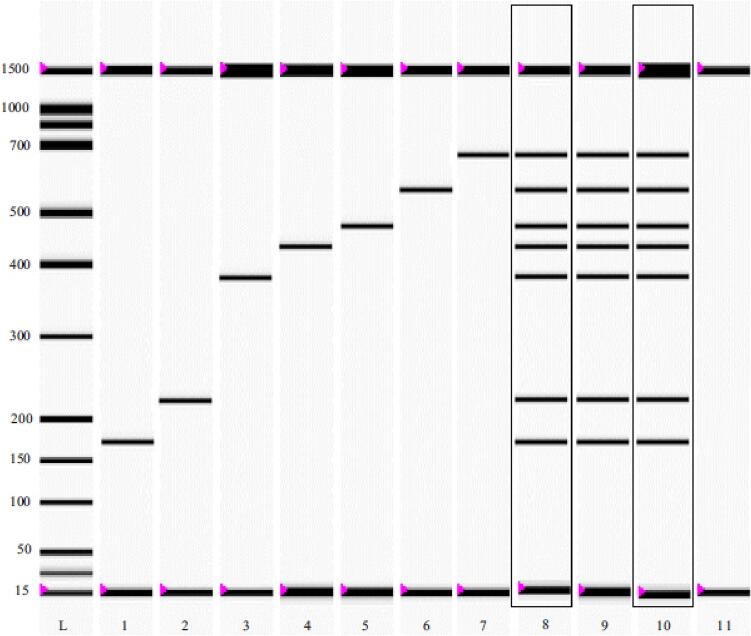
The gel image and the electropherograms of multiplex PCR for the detection of *C. livia, C. moneduloides, G. gallus, C. japonica, P. colchicus, S. camelus*, and *M. gallopavo* meats in model and deliberately adulterated beef meatball under raw and processed states. In the gel image L, ladder; lanes 1–7 *C. livia, C. moneduloides, G. gallus, C. japonica, P. colchicus, S. camelus*, and *M. gallopavo* meat. Lanes 8–10 multiplex PCR products of 7 target species in model meatballs. Lane 11 is beef meatball.

Using 19 voucher samples of meat (of the 7 targets) from various sources, the assay's repeatability was evaluated. All target meat samples produced the anticipated PCR results, illustrated in Fig. 2. Meat identification was 100% accurate as a consequence of this.

Table 2 shows the findings of the Multiplex-PCR product's reproducibility. Multiplex-PCR was carried out using raw meat samples, showing the validity of the suggested approach. Two users repeated the test twenty times using kits made by a LOT and there was 100 percent concordance between the LOTs (Table 2). The investigation was also carried out 20 times by one user, using kits from two different lots. There was 100 percent agreement between the LOTs in the results of this test (Table S1).

**Table 2:** Multiplex-PCR technique reproducibility.

Sample	Experimenter 1							Experimenter 2							Accordance rate (%)
	***C. livia***	***C. moneduloides***	***G. gallus***	***C. japonica***	***P. colchicus***	***S. camelus***	***M. gallopavo***	***C. livia***	***C. moneduloides***	***G. gallus***	***C. japonica***	***P. colchicus***	***S. camelus***	***M. gallopavo***	
1	+	+	+	+	+	+	+	+	+	+	+	+	+	+	100
2	+	+	+	+	+	+	+	+	+	+	+	+	+	+	100
3	+	+	+	+	+	+	+	+	+	+	+	+	+	+	100
4	+	+	+	+	+	+	+	+	+	+	+	+	+	+	100
5	+	+	+	+	+	+	+	+	+	+	+	+	+	+	100
6	+	+	+	+	+	+	+	+	+	+	+	+	+	+	100
7	+	+	+	+	+	+	+	+	+	+	+	+	+	+	100
8	+	+	+	+	+	+	+	+	+	+	+	+	+	+	100
9	+	+	+	+	+	+	+	+	+	+	+	+	+	+	100
10	+	+	+	+	+	+	+	+	+	+	+	+	+	+	100
11	+	+	+	+	+	+	+	+	+	+	+	+	+	+	100
12	+	+	+	+	+	+	+	+	+	+	+	+	+	+	100
13	+	+	+	+	+	+	+	+	+	+	+	+	+	+	100
14	+	+	+	+	+	+	+	+	+	+	+	+	+	+	100
15	+	+	+	+	+	+	+	+	+	+	+	+	+	+	100
16	+	+	+	+	+	+	+	+	+	+	+	+	+	+	100
17	+	+	+	+	+	+	+	+	+	+	+	+	+	+	100
18	+	+	+	+	+	+	+	+	+	+	+	+	+	+	100
19	+	+	+	+	+	+	+	+	+	+	+	+	+	+	100
20	+	+	+	+	+	+	+	+	+	+	+	+	+	+	100

#### 3.4.3 Sensitivity test of kit

To establish the test sensitivity, or the lowest quantity of mtDNA from each target species of meat that could still be identified by the assay, seven mtDNA concentrations were amplified using the multiplex assay. Each target species had a varying quantity of detectable mtDNA (Table 3). PCR products of *C. livia, C. moneduloides, G. gallus, C. japonica* were detected from as low as 12,500 mtDNA copies. PCR products of *P. colchicus* and *S. camelus* were detected from 25,000 mtDNA copies and *M. gallopavo* were detected from 50,000 mtDNA copies, respectively. Therefore, the sensitivity of the prepared kit for different meat samples was determined to be 50,000 copies/microliter to avoid false negative results.

**Table 3:** Multiplex PCR assay sensitivity analysis (+++ indicates high intensity, ++ indicates medium intensity, + indicates low intensity, and - indicates band absence).

Meat species	mtDNA amount (copies)				
	**100,000**	**50,000**	**25,000**	**12,500**	**6250**
*C. livia*	+++	++	++	+	**–**
*C. moneduloides*	+++	++	++	+	**–**
*G. gallus*	+++	++	++	+	**–**
*C. japonica*	+++	++	++	+	**–**
*P. colchicus*	++	++	+	**–**	**–**
*S. camelus*	++	++	+	**–**	**–**
*M. gallopavo*	++	+	**–**	**–**	**–**

#### 3.4.4 Regarding manufactured food products

The generated assay was used in the real world to test commercial animal products and food products. The findings for all food items are shown in Table 4. The outcome demonstrated that the multiplex PCR test was reliable and could be used. Fig. 2 displayed the electropherograms and gel images of the multiplex PCR used to identify the samples.

**Table 4:** Analysis of prototype and commercial meatballs.

*Meatballs*	Identified species							PCR accuracy (%)
	***C. livia***	***C. moneduloides***	***G. gallus***	***C. japonica***	***P. colchicus***	***S. camelus***	***M. gallopavo***	
*C. livia*	9/9	–	–	–	–	–	–	100
*C. moneduloides*	–	9/9	–	–	–	–	–	100
*G. gallus*	–	–	9/9	–	–	–	–	100
*C. japonica*	–	–	–	9/9	–	–	–	100
*P. colchicus*	–	–	–	–	9/9	–	–	100
*S. camelus*	–	–	–	–	–	9/9	–	100
*M. gallopavo*	–	–	–	–	–	–	9/9	100
Meatballs (the target raw meat species)	9/9	9/9	9/9	9/9	9/9	9/9	9/9	100
Meatballs (heated target meat species)	9/9	9/9	9/9	9/9	9/9	9/9	9/9	100
Meatballs (Beef + all raw meat target)	9/9	9/9	9/9	9/9	9/9	9/9	9/9	100
Meatballs (Beef + all raw heated target)	9/9	9/9	9/9	9/9	9/9	9/9	9/9	100
Beef Meatballs A	–	–	–	–	–	–	–	100
Beef Meatballs B	–	–	–	–	–	–	–	100
Beef Meatballs C	–	–	–	–	–	–	–	100

## 4 Discussion

The multiplex PCR test created in this work can be finished in only 90 min, doesn't need expensive equipment (such as a real-time PCR instrument or gel electrophoresis), and avoids money and time on DNA extraction (Thakur et al., 2011). For the first time, a multiplex PCR test for simultaneous meat species identification from raw meats and food items has been examined, successfully developed, and thoroughly validated (Yang et al., 2022). The test boosted the probability of target identification even in the damaged materials since the short-length nucleic acid targets are highly durable under processing circumstances, and mitochondrial genes are present in many copies (Rahman et al., 2014).

In this work, short-length amplicons in the size range 162–619 bp were created to be amplified using seven pairs of species-specific primers that target the intra-species conserved and inter-species highly variable sections of mitochondrial genes. Because the efficacy of multiplex PCR depends on the primers' capacity to selectively anneal with their particular targets under a single set of PCR circumstances, such as reaction volume, cycling, and annealing, the primer particularity and melting temperature (Tm) are extremely important (Rahman et al., 2014, Piri-Gharaghie et al., 2022b). Thus, the primer design represents a crucial stage in the development of multiplex PCR and must include sufficient intra-species conserved sequences and inter-species polymorphism with closely comparable Tm. The primers used here had extremely tight spacing between Tm (61–62 ˚C) and 5–13 bp (23–45%) mismatches with other essential species to ensure that they would only anneal with the DNA templates of the target species and not any non–target species (Tang et al., 2017).

It has also been shown that adding some substances to the PCR buffer might boost inhibitor tolerance, such as BSA and Tween-20. The buffer's composition is unknown to consumers since it is a proprietary object. Aside from that, the dilution process would have also diluted the inhibitors (Ali et al., 2015). This innovative test is inexpensive and quick since the PCR process doesn't need a comprehensive and drawn-out pre-extraction or extraction phase. It solves the previously mentioned problems with traditional PCR-based techniques. The test needed a relatively tiny quantity of meat samples (between 0.3 and 1.0 mm^2^) to obtain a successful amplification (Abdul-Hanssan and Tauma, 2014). This demonstrates that even minute quantities have enough DNA for amplification. This sensitivity was made possible by the PCR method's lack of a DNA extraction step, which prevented DNA loss (Sul et al., 2020). According to our prior experience with animal muscle samples, the ideal meat sample size identified in this research is one mm^2^, producing the maximum amplification success rate and PCR result when utilizing PCR. Small sample sizes are crucial for meat authenticity because even minute levels of contamination or fake components may affect the quality of the meat. Therefore, it is shown that the amplification approach is highly likely to identify falsifications, even at microscopic levels (Sentandreu and Sentandreu, 2014).

The food items were examined using the devised assay, and none revealed *C. moneduloides* contamination. The mitochondrial genes found in the mtDNA were used to build the primers. Three advantages result from this. First off, these genes have substantial DNA variation across various species and minimal DNA variation between individuals of identical species, giving them great confidence in animal species discrimination. In other words, the specificity is not compromised by the short amplicon length. Second, the test is exceptionally reliable and sensitive due to the roughly 3500 mitochondrial copies that may be found in a single muscular cell. Last, the sequence information from mitochondrial genes is accessible on DNA databases for numerous species, enabling further sequencing comparisons if necessary. Because it is quicker and less expensive than barcoding, the species-specific primer approach used in this study is superior to barcoding (Chen et al., 2012). The findings are simpler to understand but can only be applied to species covered by the multiplex. The seven species chosen include a mix of bird meats that will benefit international food safety organizations. The multiplex in this research adds four extra targets compared to the previously reported triplex test (Kitpipit et al., 2013). Further confidence in the assay's outcomes is gained by increasing the sample sizes for specificity, reproducibility, and commercial goods (Kitpipit et al., 2014; Smith, 2021).

There are two restrictions on the suggested assay. First, unlike real-time PCR, the experiment cannot offer quantitative data. However, due to the possibility of the food matrix interfering with the amplification process, the reliable measurement for real-time PCR could only be accomplished when an adequate reference material is employed (Smith, 2021). In other words, since there may be elements in the cooking procedure that are co-extracted with the DNA and interact with the quantification process, serially diluted DNA from raw meats cannot be utilized as a reference for DNA obtained from food samples. The second restriction is the seven species' different amplification efficiencies and sensitivities; however, this issue arises in all multiplex PCR tests (Moffa et al., 2020, Doosti et al., 2014).

The assay's precision, specificity, sensitivity, and suitability for analyzing food samples have all been confirmed. The test was shown to yield 100 percent accuracy in identifying the seven types of meat that were received from various sources. Since it only applies to the target meat species, there is no potential for mistakes or misidentification. As a result of the assay's high sensitivity, which can identify samples with as little as 50,000 mtDNA copies or seven femtograms of mtDNA (equivalent to less than seven muscle cells), thermally processed food samples may be successfully tested. This sensitivity is comparable to published traditional and real-time singleplex and multiplex PCR assays, such as the 300-cell assay (Murugaiah et al., 2009), the ten-cell assay (Köppel et al., 2008, Kargar et al., 2013), and the single-cell assay, which were all calculated from a single sample of 0.0035 ng of DNA. It is difficult to make comparisons with several research since they reported test sensitivity as a percentage of a combined sample. The robustness of the improved multiplex test to identify different meat fraud schemes in actual food samples is also shown. These data show that the developed multiplex test can successfully identify seven different types of meat from various dietary samples.

## 5 Conclusion

Identifying meat species in raw meats and food products has never been done using a multiplex PCR. *Columba livia, Corvus moneduloides, Gallus gallus, Coturnix japonica, Phasianus colchicus, Struthio camelus, and Meleagris gallopavo* are the seven widely eaten meat species that the test has successfully been designed to identify concurrently. The assay has also undergone thorough validation to fulfill the requirements for usage in actual law enforcement. Detecting adulterations and misbranding in a range of meats and food products, including highly processed or deteriorated food samples, is accurate, precise, sensitive, and relevant.

## Declaration of Competing Interest

The authors declare that they have no known competing financial interests or personal relationships that could have appeared to influence the work reported in this paper.

## Data Availability

Data will be made available on request.

## Appendix A Supplementary data

1
