# Supplementary material for: Designing a novel method based on multiplex PCR for detecting various meat of birds in processed ground meat products
Source: Food Chem (Oxf). 2023 Jul 13;7:100177. doi: 10.1016/j.fochms.2023.100177 (PMC10753382; doi:10.1016/j.fochms.2023.100177)
Supplement: 1 [file mmc1.docx]

**Supplementary materials**


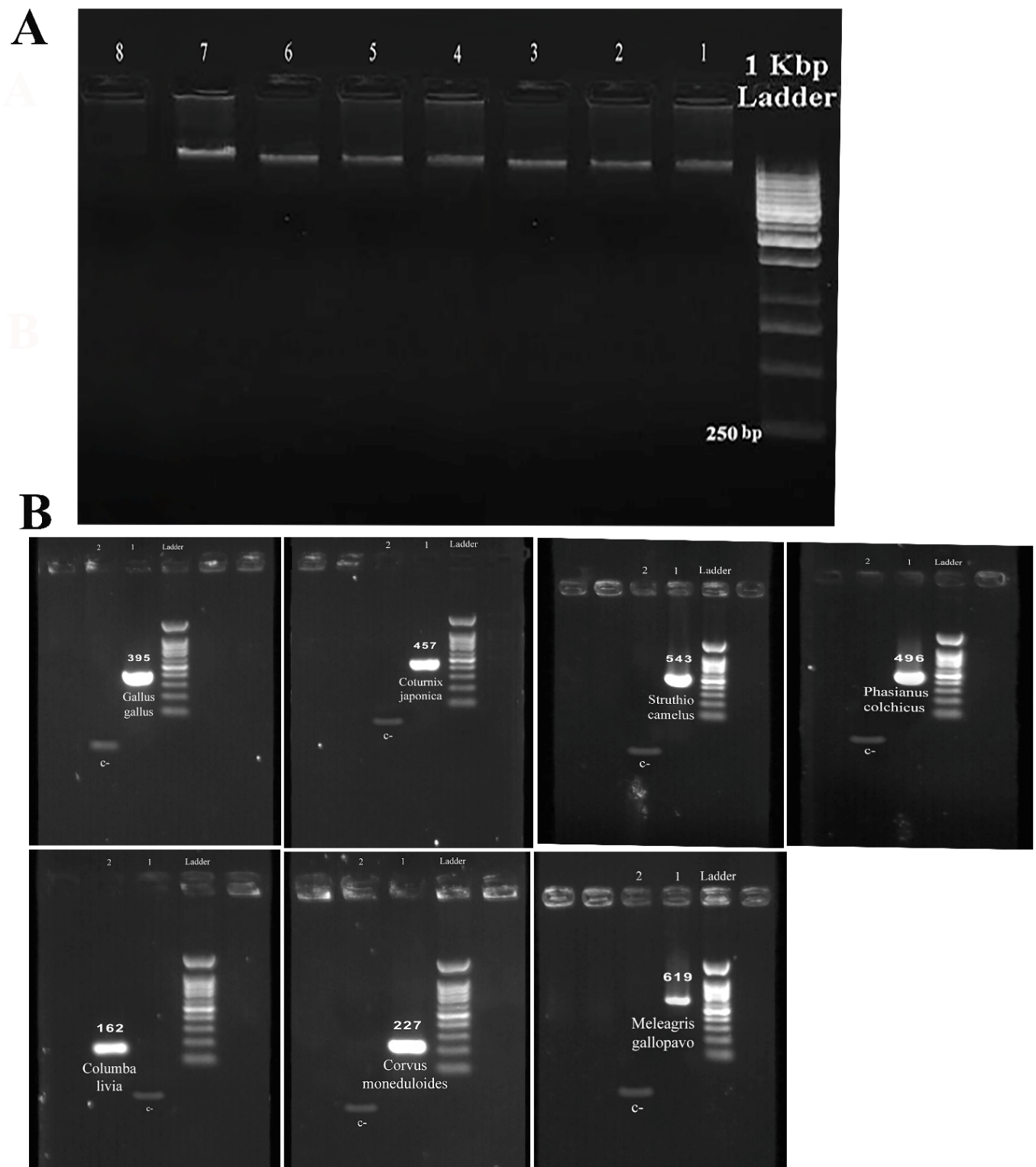


Fig S1. Seven target species isolated on a 2% agarose gel were amplified directly in a single-plex. Ladder: 100 bp, 1: *Columba livia* (162 bp), 2: *Corvus moneduloides* (227 bp), 3: *Gallus gallus* (395 bp), 4: *Coturnix japonica* (457 bp), 5: *Phasianus colchicus* (496 bp), 6: *Struthio camelus* (543 bp), 7: *Meleagris gallopavo* (619 bp), and 8: negative control. All lanes showed the anticipated PCR products.

**Table S1** Multiplex-PCR technique reproducibility

| Sample | LOT1 | | | | | | | LOT2 | | | | | | | Accordance rate (%) |
| --- | --- | --- | --- | --- | --- | --- | --- | --- | --- | --- | --- | --- | --- | --- | --- |
|  | ***C. livia*** | ***C. moneduloides*** | ***G. gallus*** | ***C. japonica*** | ***P. colchicus*** | ***S. camelus*** | ***M. gallopavo*** | ***C. livia*** | ***C. moneduloides*** | ***G. gallus*** | ***C. japonica*** | ***P. colchicus*** | ***S. camelus*** | ***M. gallopavo*** |  |
| 1 | + | + | + | + | + | + | + | + | + | + | + | + | + | + | 100 |
| 2 | + | + | + | + | + | + | + | + | + | + | + | + | + | + | 100 |
| 3 | + | + | + | + | + | + | + | + | + | + | + | + | + | + | 100 |
| 4 | + | + | + | + | + | + | + | + | + | + | + | + | + | + | 100 |
| 5 | + | + | + | + | + | + | + | + | + | + | + | + | + | + | 100 |
| 6 | + | + | + | + | + | + | + | + | + | + | + | + | + | + | 100 |
| 7 | + | + | + | + | + | + | + | + | + | + | + | + | + | + | 100 |
| 8 | + | + | + | + | + | + | + | + | + | + | + | + | + | + | 100 |
| 9 | + | + | + | + | + | + | + | + | + | + | + | + | + | + | 100 |
| 10 | + | + | + | + | + | + | + | + | + | + | + | + | + | + | 100 |
| 11 | + | + | + | + | + | + | + | + | + | + | + | + | + | + | 100 |
| 12 | + | + | + | + | + | + | + | + | + | + | + | + | + | + | 100 |
| 13 | + | + | + | + | + | + | + | + | + | + | + | + | + | + | 100 |
| 14 | + | + | + | + | + | + | + | + | + | + | + | + | + | + | 100 |
| 15 | + | + | + | + | + | + | + | + | + | + | + | + | + | + | 100 |
| 16 | + | + | + | + | + | + | + | + | + | + | + | + | + | + | 100 |
| 17 | + | + | + | + | + | + | + | + | + | + | + | + | + | + | 100 |
| 18 | + | + | + | + | + | + | + | + | + | + | + | + | + | + | 100 |
| 19 | + | + | + | + | + | + | + | + | + | + | + | + | + | + | 100 |
| 20 | + | + | + | + | + | + | + | + | + | + | + | + | + | + | 100 |
